# Treatment Outcomes in Patients With Metastatic Renal Cell Carcinoma With Sarcomatoid and/or Rhabdoid Dedifferentiation After Progression on Immune Checkpoint Therapy

**DOI:** 10.1093/oncolo/oyad302

**Published:** 2023-11-30

**Authors:** Andrew W Hahn, Devaki Shilpa Surasi, Paul V Viscuse, Tharakeswara K Bathala, Andrew J Wiele, Matthew T Campbell, Amado J Zurita, Amishi Y Shah, Eric Jonasch, Jianjun Gao, Sangeeta Goswami, Omar Alhalabi, Priya Rao, Kanishka Sircar, Nizar M Tannir, Pavlos Msaouel

**Affiliations:** Department of Genitourinary Medical Oncology, The University of Texas MD Anderson Cancer Center, Houston, TX, USA; Department of Nuclear Medicine, Division of Diagnostic Imaging, The University of Texas MD Anderson Cancer Center, Houston, TX, USA; Division of Hematology/Oncology, Department of Internal Medicine, University of Virginia Cancer Center, University of Virginia, Charlottesville, VA, USA; Department of Nuclear Medicine, Division of Diagnostic Imaging, The University of Texas MD Anderson Cancer Center, Houston, TX, USA; Department of Hematology/Oncology, Edward-Elmhurst Medical Group, Elmhurst, IL, USA; Department of Genitourinary Medical Oncology, The University of Texas MD Anderson Cancer Center, Houston, TX, USA; Department of Genitourinary Medical Oncology, The University of Texas MD Anderson Cancer Center, Houston, TX, USA; Department of Genitourinary Medical Oncology, The University of Texas MD Anderson Cancer Center, Houston, TX, USA; Department of Genitourinary Medical Oncology, The University of Texas MD Anderson Cancer Center, Houston, TX, USA; Department of Genitourinary Medical Oncology, The University of Texas MD Anderson Cancer Center, Houston, TX, USA; Department of Genitourinary Medical Oncology, The University of Texas MD Anderson Cancer Center, Houston, TX, USA; Department of Genitourinary Medical Oncology, The University of Texas MD Anderson Cancer Center, Houston, TX, USA; Department of Pathology, Division of Pathology/Lab Medicine, The University of Texas MD Anderson Cancer Center, Houston, TX, USA; Department of Pathology, Division of Pathology/Lab Medicine, The University of Texas MD Anderson Cancer Center, Houston, TX, USA; Department of Genitourinary Medical Oncology, The University of Texas MD Anderson Cancer Center, Houston, TX, USA; Department of Genitourinary Medical Oncology, The University of Texas MD Anderson Cancer Center, Houston, TX, USA; Department of Translational Molecular Pathology, The University of Texas MD Anderson Cancer Center, Houston, TX, USA; David H. Koch Center for Applied Research of Genitourinary Cancers, The University of Texas, MD Anderson Cancer Center, Houston, USA

**Keywords:** sarcomatoid, rhabdoid, cabozantinib, immune checkpoint therapy, renal cell carcinoma, somatic DNA

## Abstract

**Background:**

Metastatic RCC with sarcomatoid and/or rhabdoid (S/R) dedifferentiation is an aggressive disease associated with improved response to immune checkpoint therapy (ICT). The outcomes of patients treated with VEGFR-targeted therapies (TT) following ICT progression have not been investigated.

**Patients and Methods:**

Retrospective review of 57 patients with sarcomatoid (S), rhabdoid (R), or sarcomatoid plus rhabdoid (S + R) dedifferentiation who received any TT after progression on ICT at an academic cancer center. Clinical endpoints of interest included time on TT, overall survival (OS) from initiation of TT, and objective response rate (ORR) by RECIST version 1.1. Multivariable models adjusted for epithelial histology, IMDC risk, prior VEGFR TT, and inclusion of cabozantinib in the post-ICT TT regimen.

**Results:**

29/57 patients had S dedifferentiation and 19 had R dedifferentiation. The most frequently used TT was cabozantinib (43.9%) followed by selective VEGFR TT (22.8%). The median time on TT was 6.4 months for all, 6.1 months for those with S dedifferentiation, 15.6 months for R dedifferentiation, and 6.1 months for S + R dedifferentiation. Median OS from initiation of TT was 24.9 months for the entire cohort, and the ORR was 20.0%. Patients with R dedifferentiation had significantly longer time on TT than those with S dedifferentiation (HR 0.44, 95% CI, 0.21-0.94). IMDC risk was associated with OS.

**Conclusions:**

A subset of patients with S/R dedifferentiation derive clinical benefit from TT after they have progressive disease on ICT. Patients with R dedifferentiation appeared to derive more benefit from TT than those with S dedifferentiation.

## Teaser

As more patients with sarcomatoid and/or rhabdoid dedifferentiation experience clinical benefit using first-line immune checkpoint therapy (ICT) combinations, there is a growing need to understand treatment patterns and outcomes after progression on ICT. This article reports clinical outcomes of patients with metastatic renal cell carcinoma and sarcomatoid and/or rhabdoid dedifferentiation treated with targeted therapy after progression on ICT.

**Implications for Practice**

Targeted therapies (TT) only benefit a subset of patients with metastatic RCC with sarcomatoid and/or rhabdoid dedifferentiation whose cancer worsens on ICT. More specifically, those patients with rhabdoid dedifferentiation had significantly longer time on TT than those with sarcomatoid dedifferentiation

## Introduction

The prognosis for patients diagnosed with metastatic renal cell carcinoma (RCC) is primarily driven by clinicopathologic factors.^1^ Clinical factors are currently summarized by the International Metastatic RCC Database Consortium (IMDC) risk score that categorizes patients into favorable, intermediate, or poor risk disease.^2^ Pathologic determinants of prognosis for metastatic RCC are primarily tumor histology and dedifferentiation status. Approximately, 75% of metastatic RCC cases are clear cell histology (ccRCC), where the clear appearance is driven by accumulation of lipid droplets.^3^ Variant histologies of RCC include papillary RCC, chromophobe RCC, renal medullary carcinoma (RMC), other molecularly defined RCCs, and unclassified RCCs.^4^ Each of these histologic subtypes has distinct biologic hallmarks, and any histologic subtype may have dedifferentiation present to varying degrees.^5^ RCC may have 2 types of dedifferentiation present, sarcomatoid, and/or rhabdoid dedifferentiation.^6^ Dedifferentiation is also referred to as “differentiation” or “features” in the literature, and patients can have varying degrees of dedifferentiation present, ranging from 5%-100% of the tumor.^6^ Dedifferentiation is an uncommon event that carries a poor prognosis, and either sarcomatoid and/or rhabdoid dedifferentiation is considered World Health Organization (WHO)/International Society of Urological Pathology (ISUP) grade 4.^7^ Sarcomatoid dedifferentiation consist of atypical spindle cells that resemble a sarcoma, and it is most commonly observed in ccRCC (5% of ccRCC cases) and chromophobe RCC (8% of chromophobe cases).^8-10^ The sarcomatoid component of an RCC is thought to arise from a common cell of origin within the epithelial component.^11,12^ Rhabdoid dedifferentiation is defined by the appearance of cancer cells that resemble rhabdomyoblasts and is thought to be present in ~5% of RCC cases, most commonly in ccRCC.^13,14^ Tumors also may have both sarcomatoid and rhabdoid dedifferentiation present.

The presence of dedifferentiation has therapeutic implications driven by its unique biology and aggressive clinical course. Historically, sarcomatoid dedifferentiation was associated with poor outcomes using systemic therapies developed for metastatic ccRCC. Between 2005 and 2015, VEGF and mTOR-targeted therapy (TT) were the primary treatments for metastatic RCC.^15,16^ These agents were investigated in patients with sarcomatoid dedifferentiation alone or in combination with chemotherapy, but clinical outcomes were disappointing with median time to progression of ~5 months and objective response rates (ORR) of ~20%.^6,17-19^ More recently, immune checkpoint therapy (ICT) meaningfully improved outcomes for patients with metastatic RCC and sarcomatoid dedifferentiation.

In a post hoc analysis of CheckMate-214, nivolumab plus ipilimumab produced an ORR of 61% with 19% of patients experiencing a complete response (CR) in those with metastatic clear cell RCC and sarcomatoid dedifferentiation, and this translated to an improvement in progression-free survival (PFS; HR 0.54, 95% CI, 0.33-0.86, median 26.5 vs. 5.1 months) and overall survival (OS; HR 0.45, 95% CI, 0.30-0.70, median NR vs. 14.2 months).^20^ First-line combination of ICT plus TT have also improved clinical endpoints when compared to VEGF TT alone.^21-24^ There is biologic rationale for the dramatic responses observed with ICT in patients who had sarcomatoid dedifferentiation because sarcomatoid dedifferentiation has increased expression of PD-L1 and an inflamed tumor microenvironment.^25-29^ Less is known about clinical outcomes with systemic therapy for rhabdoid dedifferentiation, but preliminary data suggests that ICT combinations also produce meaningful clinical benefit in rhabdoid dedifferentiation.^28,30^ Unfortunately, most RCCs with sarcomatoid or rhabdoid dedifferentiation who initially respond to ICT will ultimately develop resistance and require subsequent treatment options.^20-23^

As more patients with sarcomatoid and/or rhabdoid dedifferentiation experience clinical benefit using first-line ICT combinations, there is a growing need to understand treatment patterns and outcomes after progression on ICT. In this setting, patients with metastatic RCC are typically treated with multikinase TT agents, such as cabozantinib or lenvatinib plus the mTOR inhibitor, everolimus, or selective VEGFR TT. Herein, we report clinical outcomes of patients with metastatic RCC and sarcomatoid and/or rhabdoid dedifferentiation treated with TT after progression on ICT.

## Methods

We retrospectively reviewed the records of patients with mRCC and sarcomatoid, rhabdoid, or sarcomatoid plus rhabdoid dedifferentiation who experienced progressive disease on an ICT-based treatment regimen and subsequently received a TT at the University of Texas MD Anderson Cancer Center (MDACC) between November 2015 and April 2021. Patients were identified by electronic medical record search for “sarcomatoid” or “rhabdoid” and “renal cell carcinoma,” and eligible patients had metastatic disease and progressive disease on an ICT-based regimen. In order to define presence of dedifferentiation, a patient’s pathology had to be reviewed by one of the genitourinary pathologists at MDACC for routine clinical care and reported as having dedifferentiation present. Patients with any degree of dedifferentiation were included in the cohort, and over the years included, our pathologists have variably used categorical presence (yes/no) and degree of dedifferentiation when reporting clinical pathology for dedifferentiation. Given the retrospective nature of this study, the samples were not rereviewed by independent, blinded pathologists. The type of TT after receipt of ICT was categorized as cabozantinib monotherapy, lenvatinib plus everolimus, selective VEGFR targeted therapy such as axitinib, pazopanib, or sunitinib, or ICT plus a TT such as cabozantinib, lenvatinib, or axitinib. The institutional review board approved this study. Clinical data were collected by individual chart review from the electronic medical record systemic. Demographic characteristics, histological subtype, International Metastatic RCC Database Consortium (IMDC) risk score at initiation of TT, sites of disease, and prior systemic therapies were recorded.

The clinical endpoints of interest were time on TT and overall survival (OS) from initiation of TT. Time on TT was calculated as the time from TT initiation until discontinuation for any reason, and patients who remained on TT at the time of analysis were censored using the date of last follow up. OS was calculated as the time from TT initiation until death or last follow up, if a patient was still living at the time of data collection. Two board-certified radiologists (D.S.S. and T.K.B.), blinded to clinical data, assessed radiographic tumor response using RECIST version 1.1.^31^ For each patient, one of these radiologist performed RECIST measures, so they were not confirmed by the other radiologist. Patients without baseline scans or without measurable lesions at baseline were considered not evaluable (NE). Directed acyclic graphs (DAGs) were used to avoid collider bias and identify potential confounders and mediators to be adjusted in multivariable Cox regression models when estimating the total and directed effects of exposures of interest.^32^ We prespecified type of dedifferentiation and IMDC risk score, as the 2 exposures of interest to assess their impact on OS and time on TT (Supplementary Fig. S1A-S1B). The multivariable analysis for type of dedifferentiation adjusted for histology as a confounding variable, and it did not adjust for mediators in Supplementary Fig. S1A. The multivariable analysis for IMDC risk score adjusted for histology and type of dedifferentiation as confounding variables, and it did not adjust for mediators in Supplementary Fig. S1B. Median survival times were calculated using the Kaplan-Meier method. Follow-up time was calculated based on the reverse Kaplan-Meier method. Hazard ratios (HR) and 95% confidence intervals (CI) were estimated using multivariable Cox regression models.

## Results

Between November 2015 and April 2021, 57 patients initiated a TT after progressing on an ICT-based regimen. The median follow-up time was 49.9 months. Sarcomatoid was the most common type of dedifferentiation present (*n* = 29) followed by rhabdoid (*n* = 19) and then mixed sarcomatoid and rhabdoid dedifferentiation (*n* = 9). Histologic subtype by type of dedifferentiation is reported in Table 1. Some patients with sarcomatoid dedifferentiation had chromophobe (*n* = 2/29) or unclassified histology (*n* = 3/29), but every patient with rhabdoid or mixed sarcomatoid and rhabdoid dedifferentiation had clear cell histology. Prior to receipt of TT, 48 patients (84.2%) had previously underwent a nephrectomy, and 34 patients (59.6%) received metastasis-directed therapy. At initiation of TT, 54.4% of patients had 2 or 3 anatomic sites with metastases present, and most patients had IMDC intermediate (66.7%) or poor risk (28.1%) disease. Prior to receipt of TT, the most common ICT-based regimen was nivolumab plus ipilimumab (42.1%) followed by ICT monotherapy (31.6%) and ICT plus VEGF TT (21.1%). Most patients (93%) discontinued ICT due to progressive disease, but 4 patients discontinued ICT due to immune-related adverse effects. The ORR with an ICT-based regimen was 36.4% (20/55) and an additional 25.5% (14/55) had stable disease as best response to ICT-based therapy. Median time on ICT-based regimen was 5.7 months (interquartile range 3.5-10.4 months).

**Table 1.** Baseline characteristics and treatment information for all patients and by type of dedifferentiation.

	Metastatic sarcomatoid RCC (*n* = 29)	Metastatic rhabdoid RCC (*n* = 19)	Metastatic S + R RCC (*n* = 9)	Full cohort (*n* = 57)
Histology				
Clear cell	24 (82.8%)	19 (100%)	9 (100%)	52 (91.2%)
Chromophobe	2 (6.9%)	0 (0%)	0 (0%)	2 (3.5%)
Unclassified	3 (10.3%)	0 (0%)	0 (0%)	3 (5.3%)
# metastases at ICT				
1	1 (3.4%)	4 (21.1%)	2 (22.2%)	7 (12.3%)
2-3	14 (48.3%)	12 (63.1%)	5 (55.6%)	31 (54.4%)
≥ 4	14 (48.3%)	3 (15.8%)	1 (11.1%)	18 (31.6%)
N/A	0 (0%)	0 (0%)	1 (11.1%)	1 (1.8%)
Prior nephrectomy				
Yes	23 (79.3%)	16 (84.2%)	9 (100%)	48 (84.2%)
No	6 (20.7%)	3 (15.8%)	0 (0%)	9 (15.8%)
Metastasis-directed therapy				
Yes	22 (75.9%)	10 (52.6%)	2 (22.2%)	34 (59.6%)
No	7 (24.1%)	9 (47.4%)	7 (77.8%)	23 (40.4%)
Prior VEGF TT				
Yes	14 (48.3%)	8 (42.1%)	4 (44.4%)	26 (45.6%)
No	15 (51.7%)	11 (57.9%)	5 (55.6%)	31 (54.4%)
Type of prior ICT				
Nivo + ipi	12 (41.4%)	9 (47.4%)	3 (33.3%)	24 (42.1%)
ICT monotherapy	12 (41.4%)	3 (15.8%)	3 (33.3%)	18 (31.6%)
ICT + VEGF TT	4 (13.8%)	6 (31.6%)	2 (22.2%)	12 (21.1%)
ICT + other	1 (3.4%)	1 (5.2%)	1 (1.1%)	3 (5.2%)
Reason for ICT d/c				
Progressive disease	26 (89.7%)	18 (94.7%)	9 (100%)	53 (93.0%)
Immune toxicity	3 (10.3%)	1 (5.3%)	0 (0%)	4 (7.0%)
IMDC risk score at TT				
Favorable	1 (3.4%)	0 (0%)	1 (11.1%)	2 (3.5%)
Intermediate	20 (69.0%)	13 (68.4%)	5 (55.6%)	38 (66.7%)
Poor	8 (27.6%)	6 (31.6%)	2 (22.2%)	16 (28.1%)
N/A	0 (0%)	0 (0%)	1 (11.1%)	1 (1.1%)
Type of TT post-ICT				
Cabozantinib	16 (55.2%)	3 (15.8%)	6 (66.7%)	25 (43.9%)
Other VEGFR TT	7 (24.1%)	5 (26.3%)	1 (11.1%)	13 (22.8%)
Len + eve	2 (6.9%)	4 (21.1%)	1 (11.1%)	7 (12.2%)
ICT + TT	4 (13.8%)	7 (36.8%)	1 (11.1%)	12 (21.1%)

Abbreviations: RCC: renal cell carcinoma; S + R: sarcomatoid plus rhabdoid; #: number of; ICT: immune checkpoint therapy; N/A: not available; VEGF: vascular endothelial growth factor; TT: targeted therapy; nivo: nivolumab; ipi: ipilimumab; d/c: discontinuation; IMDC: International Metastatic RCC Database Consortium; VEGFR: vascular endothelial growth factor receptor; len: lenvatinib; eve: everolimus.

**Figure 1.**
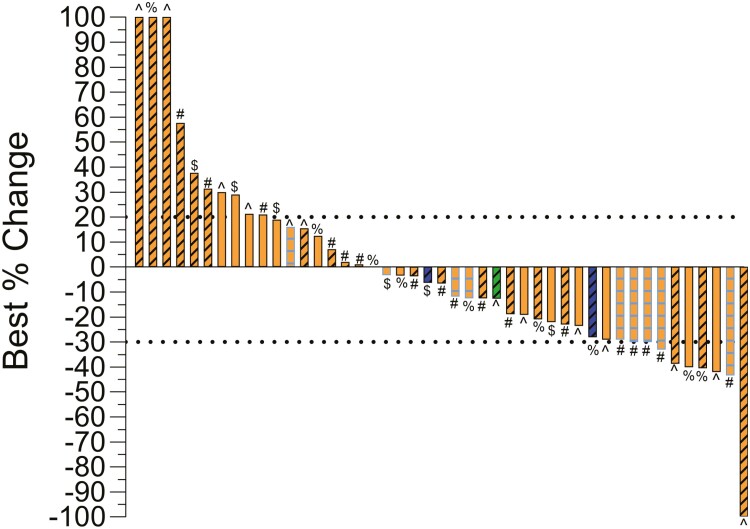
Waterfall plot of confirmed overall response. Best % change = best radiographic response of target lesion per RECIST version 1.1, as measured by a board-certified radiologist specializing in genitourinary cancers. Color represents histology. Orange is clear cell, green is chromophobe, and blue is unclassified, Shading represents type of dedifferentiation. Black diagonal is sarcomatoid, no shading is rhabdoid, and blue perpendicular is sarcomatoid and rhabdoid dedifferentiation. # is cabozantinib, % is VEGFR TT, $ is lenvatinib plus everolimus, and ^ is targeted therapy (TT) plus immune checkpoint therapy (ICT).

For type of TT after ICT, the most common treatment was cabozantinib monotherapy (43.9%) followed by selective VEGFR TT (22.8%), a TT plus ICT after progression on ICT (21.1%), and lenvatinib plus everolimus (12.2%). Selective VEGFR TT included axitinib (*n* = 10/13), pazopanib (*n* = 2/13), and sunitinib (*n* = 1/13). The TT plus ICT after progression cohort included the following treatments: cabozantinib plus nivolumab, lenvatinib plus pembrolizumab, and pembrolizumab plus axitinib. For the full cohort, the median time on TT was 6.4 months (95% CI, 4.4 to 8.5 months) and median OS from initiation of TT was 24.9 months (95% CI, 20.0 to 29.8 months). By RECIST version 1.1 criteria, ORR was noted in 9/45 patients (20.0%) with only one patient achieving a CR (Fig. 1). Stable disease was achieved in 26/45 patients (57.8%), whereas 10/45 patients (22.2%) experienced primary progressive disease. Due to lack of available imaging studies in this retrospective real-world study, 12/57 patients were NE by RECIST criteria. Since 26 patients had received TT either prior to ICT or in combination with ICT, we also evaluated ORR based on prior receipt of TT. In patients who received prior TT, the ORR was 14.3% (3/21), whereas, the ORR was 25.0% (6/24) in those who did not have prior exposure to TT.

**Figure 2.**
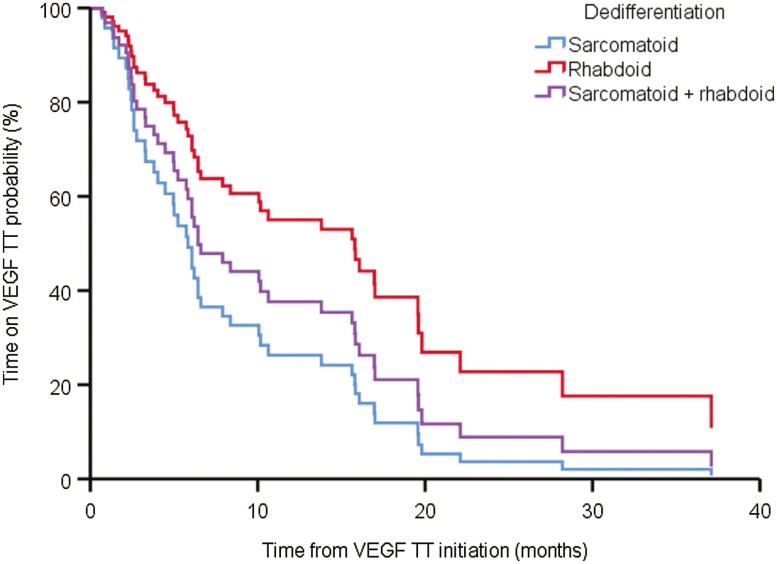
Adjusted survival curves of the direct effect of sarcomatoid and/or rhabdoid dedifferentiation on time on targeted therapy.

**Figure 3.**
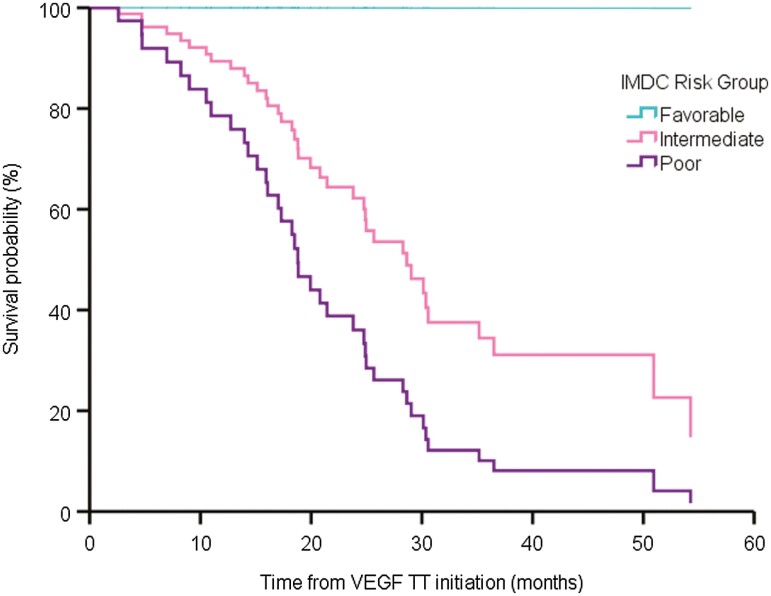
Adjusted survival curves of the direct effect of International Metastatic RCC Database Consortium (IMDC) risk score on overall survival.

Little is known about treatment outcomes with metastatic ccRCC and rhabdoid dedifferentiation, and the morphological and biological distinctions led us to hypothesize that outcomes will differ by type of dedifferentiation. In multivariable analysis, patients with metastatic RCC and rhabdoid dedifferentiation had improved time on TT after progression on ICT when compared to sarcomatoid dedifferentiation [Supplementary Table S1, hazard ratio (HR) 0.44, 95% CI, 0.21-0.94, 15.6 vs. 6.1 months, Fig. 2]. Metastatic RCC with rhabdoid dedifferentiation was not significantly associated with improved OS from TT initiation compared with sarcomatoid dedifferentiation (Supplementary Table S1). In contrast, patients with sarcomatoid plus rhabdoid dedifferentiation did not have a significant difference in time on TT or OS when compared to sarcomatoid dedifferentiation (Supplementary Table S1, Fig. 2). In a multivariable analysis, IMDC risk score was associated with OS from TT initiation (HR 2.22, 95% CI, 1.07-4.61, Fig. 3) but not time on TT (HR 1.78, 95% CI, 0.88-3.60).

**Figure 4.**
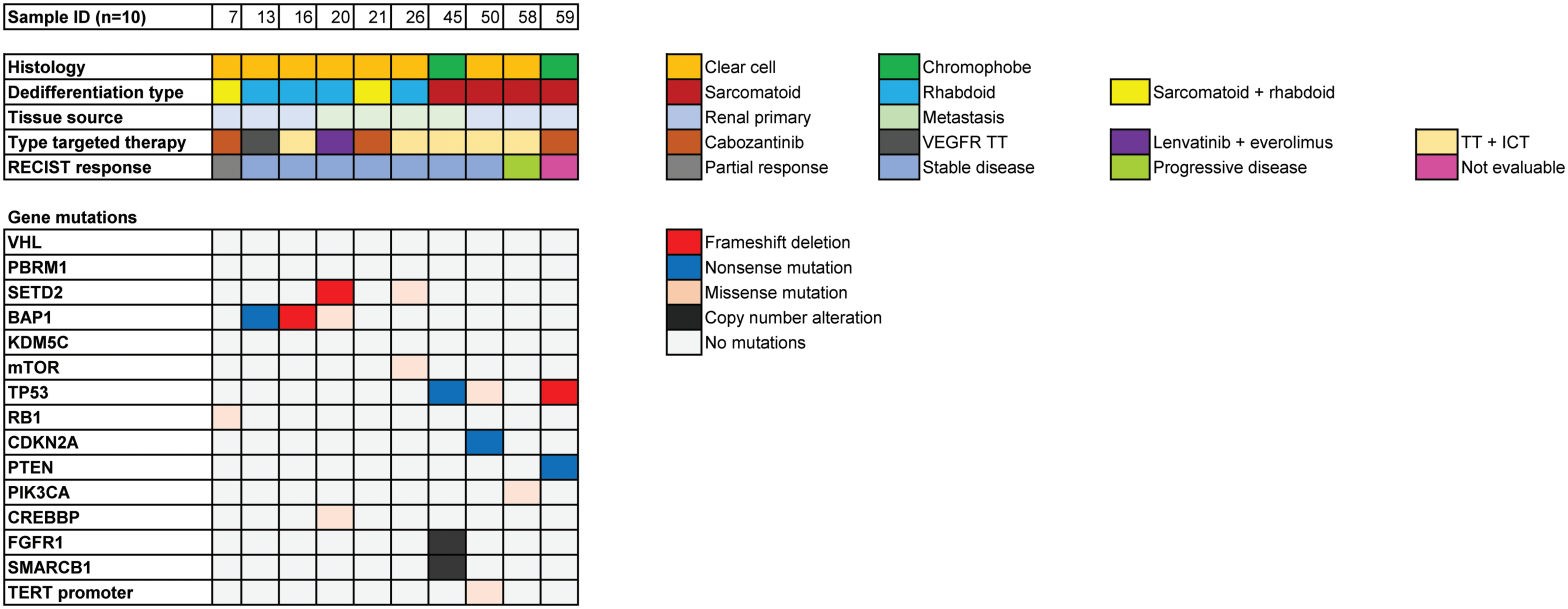
Oncoplot of somatic DNA alterations in tumor tissue from 10 patients with clinical next generation sequencing performed.

Ten patients in our cohort had clinical next generation sequencing performed (Fig. 4). For these patients, the most frequently altered genes were *BAP1*, *TP53*, and *SETD2*, whereas alterations were not observed in genes associated with favorable outcomes, such as *PBRM1* or *KDM5C*. Patients with metastatic ccRCC and rhabdoid dedifferentiation had a high frequency of genomic alterations in *BAP1* (*n* = 3/4) and *SETD2* (*n* = 2/4). The 2 patients with metastatic chromophobe RCC and sarcomatoid dedifferentiation both had genomic alterations in *TP53*.

## Discussion

The development of ICT improved outcomes for patients with metastatic RCC and sarcomatoid dedifferentiation, since these therapies have the potential to produce durable clinical responses. However, little is known about the activity of contemporary TT agents after patients with metastatic RCC and sarcomatoid and/or rhabdoid dedifferentiation develop progressive disease on ICT. In our retrospective cohort of 57 patients, a small subset of patients with metastatic RCC and sarcomatoid and/or rhabdoid dedifferentiation derived clinical benefit from TT after progression on ICT with an ORR of 20.0% and median time on TT of only 6.4 months. Despite the small sample size, patients with metastatic RCC and rhabdoid dedifferentiation had improved time on TT after progression on ICT when compared to those with sarcomatoid dedifferentiation, which is notable because the influence of rhabdoid versus sarcomatoid dedifferentiation on systemic therapy outcomes is understudied. Furthermore, we found that the IMDC risk score remains a valuable prognostic tool in patients with sarcomatoid and/or rhabdoid dedifferentiation who have received prior ICT.

Prior to the introduction of ICT, TT was investigated in multiple studies for patients with metastatic RCC and sarcomatoid dedifferentiation. Three retrospective studies found that selective VEGFR TT have limited clinical activity for patients with metastatic RCC and sarcomatoid dedifferentiation.^33-35^ In a single center study of 43 patients, VEGFR TT produced an ORR of 19% and median PFS of 5.3 months.^33^ In a subsequent IMDC database analysis, VEGFR TT produced a median PFS of 4.5 months and OS of 10.4 months.^34^ Finally, an analysis of 199 patients by our group found that survival did not improve in the TT era when compared to patients with sarcomatoid dedifferentiation who received chemotherapy or cytokine therapy.^35^ Multiple clinical trials evaluated combining VEGFR TT with chemotherapy. One of these trials randomized patients with sarcomatoid dedifferentiation to sunitinib alone versus sunitinib plus gemcitabine, and the sunitinib alone arm produced an ORR of 11.1% and a median PFS of 3.3 months.^19^ Cabozantinib and lenvatinib are multikinase TT that encompass tyrosine kinases beyond the VEGFR, such as MET, TAM receptors, and FGFR. Given the benefit of these agents verse VEGFR TT in metastatic clear cell RCC, we hoped that clinical outcomes after ICT would outperform historical controls. However, the observed ORR of 20.0% is similar to the 10%-20% reported with VEGFR TT, and the waterfall plot in Fig. 1 shows responders received cabozantinib as well as VEGFR TT. This raises an important question of what should be given to patients with sarcomatoid dedifferentiation who progress on first-line TT plus ICT, and given our findings, nivolumab plus ipilimumab warrants further investigation in this setting. A retrospective, multicenter study reported outcomes with cabozantinib as second-line or third-line therapy after prior VEGFR TT (86%) or nivolumab plus ipilimumab (14%) for patients with metastatic RCC and sarcomatoid dedifferentiation.^36^ In that study, ORR was higher at 44%-47%, median PFS was similar at 7.6 months, and median OS was shorter at 9.1 months. Our study also included a group who received TT + ICT (21.1%) after progressive disease on prior ICT, and we did not see any distinct pattern of benefit in these patients compared to multi-kinase TT alone, which aligns with the recent findings from CONTACT-03.^37^

While interest in sarcomatoid dedifferentiation increased after introduction of ICT, less remains known about systemic therapy outcomes for patients with metastatic RCC and rhabdoid dedifferentiation.

Rhabdoid and sarcomatoid dedifferentiation are both considered WHO/ISUP grade 4 tumors, and across all stages, these dedifferentiated tumors have an inferior prognosis.^7^ Thus, some clinicians view sarcomatoid and rhabdoid dedifferentiation as similar entities once metastatic. In a multiomic study of sarcomatoid and/or rhabdoid dedifferentiation, both sarcomatoid and rhabdoid dedifferentiation were found to have improved responses to ICT.^28^ Yet, prior studies suggest that sarcomatoid dedifferentiation may harbor distinct mutations from rhabdoid dedifferentiation, and our analysis of somatic DNA alterations in 10 patients demonstrated differences in *BAP1*, *SETD2*, and *TP53*.^6,38,39^ We observed a notable difference in clinical outcomes with TT after ICT between patients with sarcomatoid versus rhabdoid dedifferentiation. Patients with rhabdoid dedifferentiation had improved time on TT than those with sarcomatoid dedifferentiation and demonstrated the best percent change by RECIST. These differences suggest that the underlying biological differences between rhabdoid and sarcomatoid dedifferentiation impact treatment outcomes and will require therapeutic regimes tailored to each dedifferentiation type. Furthermore, there is an unmet need to investigate and compare the efficacy of available systemic therapies, including ICT, in large cohorts of patients sarcomatoid, rhabdoid, or mixed sarcomatoid plus rhabdoid dedifferentiation. Our findings, coupled with the histologic and biologic distinctions, suggest that sarcomatoid and rhabdoid dedifferentiation should be investigated as distinct entities in future studies.

Our study is limited by its retrospective design, the small number of patients, and the unique practice patterns of a high volume, tertiary cancer center. While our study benefited from pathologists who have expertise in the diagnosis of RCC, dedifferentiation can be heterogeneous across a tumor and at risk for sampling errors.^6^ Our study included patients who received any TT received after progression on ICT, but the small sample size did not allow us to compare the magnitude of benefit between individual TT agents.

## Conclusions

Only a subset of patients with metastatic RCC and sarcomatoid and/or rhabdoid dedifferentiation derive clinical benefit from TT after they progress on ICT. Patients with rhabdoid dedifferentiation may derive more benefit from TT than those with sarcomatoid dedifferentiation. The IMDC risk score remains a useful prognostic tool in this setting.

## Supplementary Material

Supplementary material is available at *The Oncologist* online.

oyad302_suppl_Supplementary_Figures_1

oyad302_suppl_Supplementary_Tables_1

## Data Availability

The identified data underlying this article cannot be shared publicly due to institutional compliance issues. The data will be shared on reasonable request to the corresponding author.
