# Supplementary material for: Treatment Outcomes in Patients With Metastatic Renal Cell Carcinoma With Sarcomatoid and/or Rhabdoid Dedifferentiation After Progression on Immune Checkpoint Therapy
Source: Oncologist. 2023 Nov 30;29(5):392–9. doi: 10.1093/oncolo/oyad302 (PMC11067817; doi:10.1093/oncolo/oyad302)

**Supplementary Figure 1 Legend**: DAG of the causal relationships assumed in the regression models. Arrows indicate a causal interaction between two variables. The exposures of interest are (A) dedifferentiation (sarcomatoid versus rhabdoid versus sarcomatoid plus rhabdoid and (B) International Metastatic RCC Database Consortium (IMDC) risk score. The outcomes of interest are overall survival (OS) and time on targeted therapy (TT).


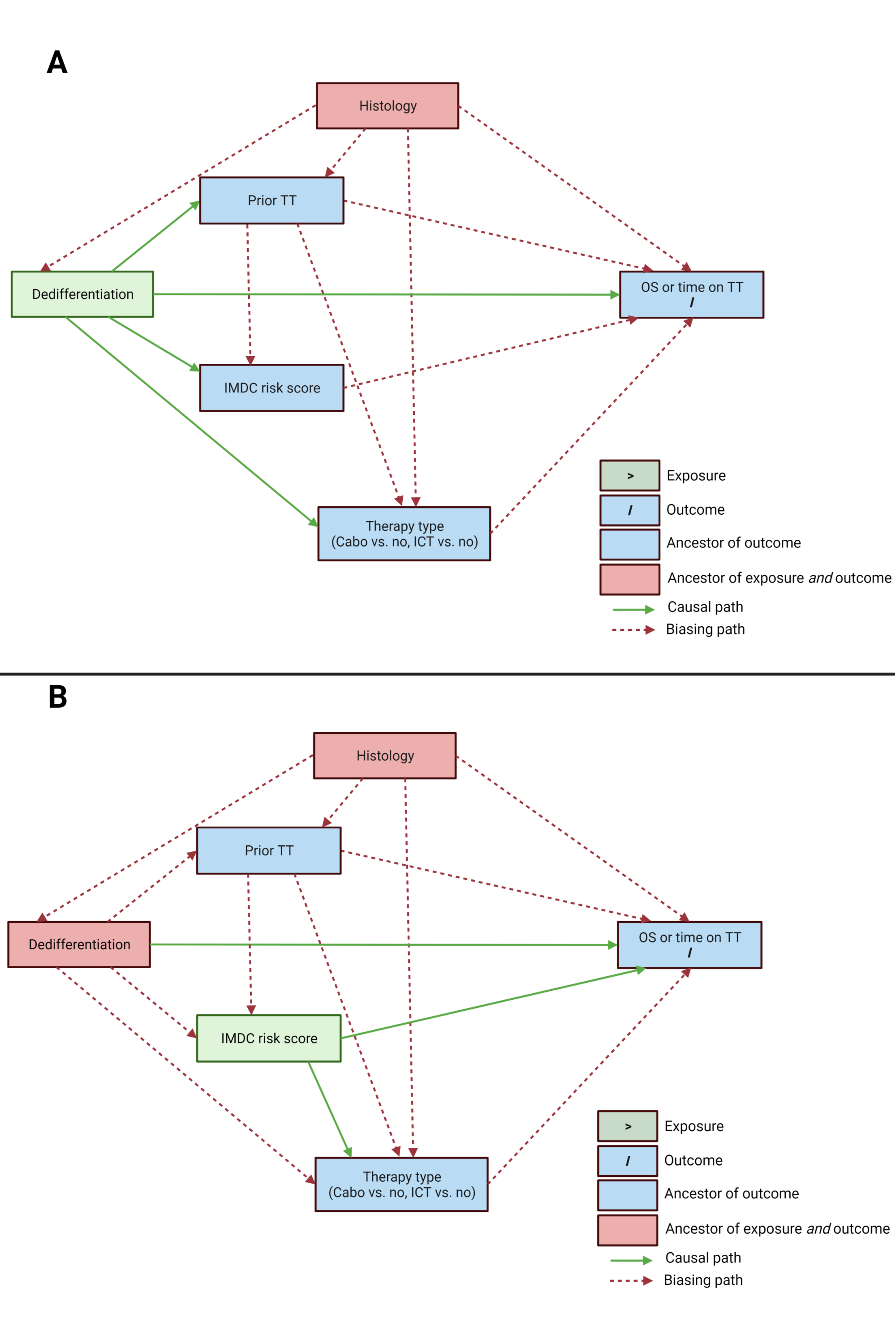

Supplement: oyad302_suppl_Supplementary_Figures_1 [file oyad302_suppl_supplementary_figures_1.docx]
