# Supplementary material for: Treatment Outcomes in Patients With Metastatic Renal Cell Carcinoma With Sarcomatoid and/or Rhabdoid Dedifferentiation After Progression on Immune Checkpoint Therapy
Source: Oncologist. 2023 Nov 30;29(5):392–9. doi: 10.1093/oncolo/oyad302 (PMC11067817; doi:10.1093/oncolo/oyad302)
Supplement: oyad302_suppl_Supplementary_Tables_1 [file oyad302_suppl_supplementary_tables_1.docx]

|  | **Metastatic sarcomatoid RCC**  **(n=29)** | **Metastatic rhabdoid RCC**  **(n=19)** | **HR** | **Metastatic S + R RCC**  **(n=9)** | **HR** |
| --- | --- | --- | --- | --- | --- |
| Median time on TT  (95% CI) | 6.1 m  (4.0 – 8.2 m) | 15.6 m  (1.1 – 30.2 m) | 0.44  (0.21 – 0.94) | 6.1 m  (5.4 – 6.8 m) | 0.73  (0.29-1.80) |
| Median OS from TT initiation  (95% CI) | 23.8 m  (16.3 – 31.1 m) | 28.6 m  (22.1 – 35.2 m) | 0.77  (0.36 – 1.62) | 35.2 m  (15.4 – 55.0 m) | 0.48  (0.16 – 1.50) |

**Supplementary Table 1 Legend**: The direct effect of dedifferentiation type on clinical outcomes with targeted therapy after progression on immune checkpoint therapy.

N = total number, RCC = renal cell carcinoma, HR = hazard ratio, S + R = sarcomatoid plus rhabdoid, TT = targeted therapy, CI = confidence interval, m = month, OS = overall survival.
